# Oil Content, Fatty Acid Composition and Distributions of Vitamin-E-Active Compounds of Some Fruit Seed Oils

**DOI:** 10.3390/antiox4010124

**Published:** 2015-01-29

**Authors:** Bertrand Matthäus, Mehmet Musa Özcan

**Affiliations:** 1 Max Rubner-Institut, Federal Research Institute for Nutrition and Food, Schützenberg 12, 32756 Detmold, Germany; E-Mail: bertrand.matthaus@mri.bund.de; 2 Department of Food Engineering, Faculty of Agriculture, University of Selcuk, 42079 Konya, Turkey

**Keywords:** fruit seed, oil content, fatty acids, vitamin-E-active compounds, by-products, oil characterisation

## Abstract

Oil content, fatty acid composition and the distribution of vitamin-E-active compounds of selected Turkish seeds that are typically by-products of the food processing industries (linseed, apricot, pear, fennel, peanut, apple, cotton, quince and chufa), were determined. The oil content of the samples ranged from 16.9 to 53.4 g/100 g. The dominating fatty acids were oleic acid (apricot seed oil, peanut oil, and chufa seed oil) in the range of 52.5 to 68.4 g/100 g and linoleic acid (pear seed oil, apple seed oil, cottonseed oil and quince seed oil) with 48.1 to 56.3 g/100 g, while in linseed oil mainly α-linolenic acid (53.2 g/100 g) and in fennel seed oil mainly 18:1 fatty acids (80.5 g/100 g) with petroselinic acid predominating. The total content of vitamin-E-active compounds ranged from 20.1 (fennel seed oil) to 96 mg/100 g (apple seed oil). The predominant isomers were established as α- and γ-tocopherol.

## 1. Introduction

Large amounts of different seeds are discarded yearly as by-products of food processing [1,2]. These seeds are often rich sources of oil and other interesting minor compounds. Since some time an increasing interest in oils from unconventional seeds has been noted [3,4,5], and the by-products generated by fruit and vegetable industry may contribute as important sources for such oils. The added value of the by-products and the potential use of these resulting seed oils depends on the fatty acid composition and the content of minor components in the oil. It is desirable that the quantities of seeds which accumulate during fruit processing as residues from various fruits and vegetables are utilized as sources for the production of oils. The continued expansion in the food industry indeed calls attention to different fruit seeds as a potential source for oil. To ensure the utilization of these by-products more information about the oil content and the composition of different fruits is required showing the economical and efficient usability of these seeds [1,6]. The aim of the present study was to compare the oil contents as well as the composition of fatty acids and vitamin-E-active compounds of oils extracted from selected seeds.

## 2. Material and Methods

### 2.1. Material

The samples were collected by hand in May and August in 2007 from plants growing at several locations of Turkey. Seeds and kernels were obtained from fruits by hand-processing and then transferred to laboratory in polypropylene bags under cool conditions. Then they were stored in glass jars at 6°C until analysis. Detailed information related to the samples is given in Table 1.

**Table 1:** Seeds used in experiment.

Sample No.	General Name	Botanical Name	Family	Locations
1	Linseed	*Linum usitatissimum*	FLinaceae	Akören-Konya
2	Apricot (sweet)	*Prunus armeniaca*	Rosacea	Beybes-Konya
3	Apricot (bitter)	*Prunus armeniaca*	Rosaceae	Beybes-Konya
4	Pear	*Pyrus communis*	Rosaceae	Ankara
5	Fennel (dulce)	*Foeniculum vulgare*	Apicaceae	Konya
6	Peanut	*Arachis hypogaea*	Leguminaceae	Silifke-Mersin
7	Apple (Golden)	*Malus communis*	Rosaceae	Eğridir-Isparta
8	Apple (Starking)	*Malus communis*	Rosaceae	Hadim-Konya
9	Cotton	*Gossypium hirsutum*	Malvaceae	Adana
10	Quince	*Cydonia vulgaris*	Malvaceae	Beybes-Konya
11	Chufa	*Cyperus esculentus*	Cyperaceae	Çumra-Konya
12	Apple (Starking)	*Malus communis*	Rosaceae	Karaman

### 2.2. Reagents

Petroleum ether (40–60) was of analytical grade (>98%; Merck, Darmstadt, Germany). Heptane and tert-butyl methyl ether were of HPLC grade (Merck, Darmstadt, Germany). Tocopherol and tocotrienol standard compounds were purchased from CalBiochem (Darmstadt, Germany).

### 2.3. Oil Content

The oil content was determined according to the method ISO 659:1998 [7]. About 2 g of the seeds were ground in a ball mill and extracted with petroleum ether in a Twisselmann apparatus for 6 h. The solvent was removed by a rotary evaporator at 40 °C and 25 Torr. The oil was dried by a stream of nitrogen and stored at −20 °C until use.

### 2.4. Fatty Acid Composition

The fatty acid composition was determined following the ISO standard ISO 5509:2000 [8]. In brief, one drop of the oil was dissolved in 1 mL of *n*-heptane, 50 μg of sodium methylate (Merck, Darmstadt, Germany) was added, and the closed tube was agitated vigorously for 1 min at room temperature. After addition of 100 μL of water, the tube was centrifuged at 4500 g for 10 min and the lower aqueous phase was removed. Then 50 μL of HCl (1 mol with methyl orange (Merck, Darmstadt, Germany)) was added, the solution was shortly mixed, and the lower aqueous phase was rejected. About 20 mg of sodium hydrogen sulphate (monohydrate, extra pure; Merck, Darmstadt, Germany) was added, and after centrifugation at 4500 g for 10 min, the top *n*-heptane phase was transferred to a vial and injected in a HP5890 gas chromotograph (Agilent Technologies Sales & Services GmbH & Co. KG, Waldbronn, Germany), with a capillary column, CP-Sil 88 (100 m long, 0.25 mm ID, film thickness 0.2 μm). The temperature program was as follows: From 155 °C; heated to 220 °C (1.5 °C/min), 10 min isotherm; injector 250 °C, detector 250 °C; carrier gas 36 cm/s hydrogen; split ratio 1:50; detector gas 30 mL/min hydrogen; 300 mL/min air and 30 mL/min nitrogen; manual injection volume less than 1 μL. The peak areas were computed by the integration software, and percentages of fatty acid methyl esters (FAME) were obtained as weight percent by direct internal normalization.

### 2.5. Vitamin-E-Active Compounds

For determination of vitamin-E-active compounds, a solution of 250 mg of oil in 25 mL of *n*-heptane was directly used for the HPLC. The HPLC analysis was conducted using a Merck-Hitachi low-pressure gradient system, fitted with a L-6000 pump (Merck-Hitachi, Darmstadt, Germany), a Merck-Hitachi F-1000 fluorescence spectrophotometer (Darmstadt, Germany; detector wavelengths for excitation 295 nm, for emission 330 nm), and a ChemStation integration system (Agilent Technologies Deutschland GmbH, Böblingen, Germany). The samples in the amount of 20 μL were injected by a Merck 655-A40 autosampler (Merck-Hitachi, Darmstadt, Germany) onto a Diol phase HPLC column 25 cm × 4.6 mm ID (Merck, Darmstadt, Germany) used with a flow rate of 1.3 mL/min. The mobile phase used was 99 mL *n*-heptane + 1 mL tert-butyl methyl ether [9]. The mean values were given in the tables, without the standard deviation, because this value would represent only the deviation of the method and not the variation of the appropriate sample.

## 3. Results and Discussion

The results for oil content and fatty acid composition of the seeds are shown in Table 2. The oil contents of the seeds were found between 16.9 g/100 g (dw) (quince) and 53.6 g/100 g (dw) (apricot seeds, sweet). While most of the samples showed oil contents below or near 30 g/100 g, the oil content of apricot seeds (sweet and bitter) was remarkably higher with showing values of 53.4 g/100 g (dw) and 45.2 g/100 g (dw), respectively. From an economical point of view, this high oil content would justify oil extraction of the seeds, whereas for seeds with oil contents below 20 g/100 g an economical extraction of oil is only meaningful by solvent when the meal contains valuable protein, e.g., soybean or if the oil can be marketed as high-quality cold-pressed edible oil. The oil content of linseed was found relatively low in comparison to the literature with 34.2 to 44.4 g/100 g [10], probably due to season, environment and locations.

Results for the fatty acid composition showed that the oils can be divided into two groups with one group predominant in 18:1 fatty acids and the other group high in linoleic acid (18:2). Only in linseed oil was α-linolenic acid predominating with amounts of 53.2 g/100 g, while its content in the other samples was between 0.1 and 1.0 g/100 g, only. The highest contents of oleic acid were found in apricot (sweet) (68.3 g/100 g), chufa (62.4 g/100 g) and apricot (bitter) (57.8 g/100 g) seed oils. Fennel (dulce) seed oil is characterized by a high content of 18:1 fatty acids (80.5 g/100 g) with petroselinic acid as predominant fatty acid. The used GLC-method was not able to separate petroselinic acid from oleic acid, but from literature it is known that petroselinic acid is predominant in members of the family Apiaceae. From a nutritional point of view this high content of oleic acid favors these seed oils for human nutrition. The content of linoleic acid of the oils ranged from 12.0 g/100 g (fennel seed oil) to 56.3 g/100 g (cotton seed oil), with half (six) of the selected seed oils comprising of at least 50 g/100 g linoleic acid. The relatively low content of saturated fatty acids such as palmitic acid or stearic acid with amounts below 10 g/100 g is interesting from a nutritional point of view. Only chufa seed oil (17.2 g/100 g) and cottonseed oil (24.2 g/100 g) had higher amounts of nutritionally unfavourable saturated fatty acids. The fatty acid composition found in the present investigation for apricot seed oil was similar to the fatty acid composition published by Dubois *et al.* [11] with 4.4 g/100 g palmitic acid, 0.5 g/100 g stearic acid, 66.3 g/100 g oleic acid, and 28.6 g/100 g linoleic acid.

The results from Dubois *et al.* [11] also confirm the presented results for chufa seed oil with 13.8 g/100 g palmitic acid, 3.2 g/100 g stearic acid, 72.6 g/100 g oleic acid, and 8.9 g/100 g linoleic acid. Similarly, Kim *et al.* [12] published the high amount of oleic acid (65.5 g/100 g) and the moderate amounts of palmitic acid (15.2 g/100 g) and linoleic acid (16.2 g/100 g) for chufa seed oil. A little different was the fatty acid composition for chufa seed oil reported by Eteshola and Oraedu [13] with 28.1 g/100 g myristic acid, 14.5 g/100 g palmitic acid, 3.4 g/100 g stearic acid, 44.8 g/100 g oleic acid and 8.8 g/100 g linoleic acids. Especially the finding of myristic acid and the low amount of linoleic acid do not agree with the results from other authors or the presented work. Different varieties or locations of cultivation can be reasons for these differences.

Reiter *et al.* [14] determined 4.4 g/100 g palmitic acid, 73.9 g/100 g petroselinic, 4.8 g/100 g oleic acid, and 16.3 g/100 g linoleic acid in fennel seed oil. The amount of 18:1 fatty acids found in the present investigation was very similar to the results from Reiter *et al.* [14]*.*

**Table 2:** Oil content and fatty acid composition of seed oils (g/100 g).

	Oil	16:0	16:1-δ-7	16:1-δ-9	18:0	Sum 18:1	18:1-δ-9	18:1-δ-11	18:2-δ-9,12	20:1-δ-11	18:3-δ-9,12,15	18:4-δ-6,9,12,15
**Linseed**	33.6	4.9	n.d.	0.1	4.8	n.d.	20.2	0.6	15.0	0.4	53.2	n.d.
**Apricot (sweet)**	53.4	4.9	n.d.	0.6	1.1	n.d.	68.3	1.4	23.1	0.1	0.1	0.1
**Apricot (bitter)**	45.2	6.4	1.0	0.9	1.0	n.d.	57.8	1.8	31.4	0.1	0.2	0.1
**Pear (Ankara)**	31.7	9.0	n.d.	0.2	2.1	n.d.	31.8	0.5	53.6	1.2	0.4	0.4
**Fennel (Dulce)**	18.2	4.3	0.2	0.2	1.6	80.5			12.0	0.3	0.4	n.d.
**Peanut**	38.3	9.5	n.d.	0.1	3.2	n.d.	52.5	n. d.	28.3	1.5	0.1	1.0
**Apple (Golden)**	21.9	7.0	0.1	0.1	1.9	n.d.	35.7	0.5	51.7	1.1	0.6	0.5
**Apple (Starking)**	25.6	6.8	0.1	0.10	2.0	n.d.	40.4	n. d.	48.1	1.2	0.3	0.5
**Cotton**	27.0	21.9	n.d.	0.5	2.3	n.d.	14.6	0.7	56.3	0.3	0.2	0.1
**Quince**	16.9	6.8	0.1	0.2	1.5	n.d.	33.8	0.6	55.4	0.6	0.2	0.3
**Chufa**	17.3	14.5	n.d.	0.1	2.7	n.d.	62.4	0.9	17.0	0.6	0.6	0.3
**Apple (Starking)**	23.5	6.3	0.1	0.1	2.1	n.d.	38.8	0.3	49.6	1.3	0.3	0.5

16:0, palmitic acid; 16:1-δ-7, 7c-hexadecenoic acid; 16:1-δ-9, 9c-hexadecenoic acid 18:0, stearic acid; Sum 18:1, 18:1-δ-6 (petroselinic acid) + 18:1-δ-9 (oleic acid) + 18:1-δ-11 (*cis*-vaccenic acid); 18:1-δ-9, oleic acid; 18:1-δ-11, *cis*-vaccenic acid; 18:2-δ-9,12, linoleic acid; 20:1-δ-11, gondoic acid; 18:3-δ-9,12,15, α-linolenic aicd; 18:4-δ-6,9,12,15, stearidonic acid; n.d., not detectable.

In linseed oil, the major fatty acids were reported as 5.5 ±1.5 g/100 g palmitic acid, 3.5 ±1.2 g/100 g stearic acid, 22.1 ±5 g/100 g oleic acid, 20.5 ±1.5 g/100 g linoleic acid and 47.5 ±5.6 g/100 g α-linolenic acid [15]. Also, Seher and Gundlach [16] established 6.7 g/100 g palmitic acid, 3.42 g/100 g stearic acid, 16.8 g/100 g oleic acid, 16.5 g/100 g linoleic acid and 54.7 g/100 g α-linolenic acid in linseed oil. Other authors found 4.0–7.0 g/100 g palmitic acid, 2.0–4.0 g/100 g stearic acid, 14.0–38.0 g/100 g oleic acid, 7.0–19.0 g/100 g linoleic acid and 35.0–66.0 g/100 g α-linolenic acid [17], while Overeem *et al.* [18] reported 5.3 g/100 g palmitic acid, 3.1 g/100 g stearic acid, 18.1 g/100 g oleic acid, 15.2 g/100 g linoleic acid and 54.10 g/100 g α-linolenic acid in linseed oil. Ryan *et al.* [19] determined 6.6 g/100 g palmitic acid, 4.1 g/100 g stearic acid, 24.0 g/100 g oleic acid, 19.90 g/100 g linoleic acid and 43.3 g/100 g α-linolenic acids. Within a certain variation the published results agree with the results of the present paper considering linseed oil as an important source of polyunsaturated α-linolenic acid with comparable low amounts of saturated fatty acids.

Palmitic acid and linoleic acid were the dominant fatty acids, constituting 70–80 g/100 g of the total fatty acids in the apple fruit [20]. As seen in Table 2, the fatty acid composition of apple seed oils in this investigation show similar results.

For peanut oil Dubois *et al.* [11] found 11.2–16.1 g/100 g palmitic acid, 3.2–4.1 g/100 g stearic acid, 35.9–58.7 g/100 g oleic acid, 20.7–37.3 g/100 g linoleic acid and 1.2–1.7 g/100 g eicosanoic acid which is in the range observed in the presented results. Other authors reported 6.0–16.0 g/100 g palmitic acid, 1.3–6.5 g/100 g stearic acid, 35.0–72.0 g/100 g oleic acid and 13.0–45.0 g/100 g linoleic acids for peanut oil [21].

In cottonseed oil, 21.0 and 27.2 g/100 g palmitic acid, 1.8 and 2.2 g/100 g stearic acid, 16.9 and 16.9 g/100 g oleic acid, 50.5 and 56.7 g/100 g linoleic acid and 0.9 and 1.2 g/100 g α-linolenic acids were determined [16], statistically within the range observed in the current study. According to Yazıcıoğlu and Karaali [21] cottonseed oil contained 17.0–31.0 g/100 g palmitic acid, 13.0–44.0 g/100 g oleic acid and 33.0–59.0 g/100 g linoleic acids. Also, Dubois *et al.* [11] determined 24.2 g/100 g palmitic acid, 2.3 g/100 g stearic acid, 17.4 g/100 g oleic acid and 53.2 g/100 g linoleic acids in cottonseed oil.

In vegetable oils four different derivatives of tocopherols and tocotrienols (α-, β-, γ-, δ-), respectively, can be found as vitamin-E-active compounds differing in the methylation of the chroman ring. Vitamin-E-active compounds have two functions: On one side they stabilize the oil against oxidative deterioration during storage or heat-treatment and on the other side they have some biological effects to humans. The main biochemical function of the tocopherols is believed to be the protection of polyunsaturated fatty acids against peroxidation [22,23]. The biological effect is derived from the inhibitory effect of vitamin-E-active compounds on the oxidation of low-density lipoprotein which may be responsible for the formation of atherosclerotic plaque, one reason for myocardial infarction and cardiovascular death [24]. Whereas the antioxidant and the anti-inflammatory properties of α-tocopherol have been well established, it is becoming increasingly evident that other isomers, especially γ-tocopherol are equally potent or possess additional biological properties [25,26]. Recent research has also shown that γ-tocopherol is a better negative risk factor for certain types of cancer and myocardial infarctions than α-tocopherol, whereas a high supplementation of α-tocopherol can deplete the body of γ-tocopherol [27].

In general the antioxidant activity increases for tocopherols and tocotrienols in the order α to δ, whereas the biological activity is opposite to the antioxidant activity [28,29]. Another member of this class of compounds is plastochromanol-8 (P-8). Its structure is similar to that of γ-tocopherol, but its basic molecular structure is a chromanol-6 ring in which methyl groups are located at positions 2, 7 and 8. The number of isoprene units on the side chain of position 2 is variable. Plastochromanol-8 is described as being more effective against oxidative deteriorations than α-tocopherol [30], while tocotrienols are less helpful in inhibiting autoxidation than tocopherols [31].

Due to the antioxidant properties the content and the composition of vitamin-E-active compounds is an important feature for the assessment of seed oils. The total content of vitamin-E-active compounds in the different seeds varied between 20.1 (fennel) and 94.0 mg/100g (quince) (Table 3). In comparison, the total content of vitamin-E-active compounds in rapeseed oil ranges between 43 and 268.0 mg/100 g and in soybean oil between 60.0 and 337.0 mg/100 g [32].According to this, the contents of vitamin-E-active compounds in the different seed oils from fruits and vegetables could be a good source for antioxidants.

**Table 3:** Distribution of vitamin-E-active compounds of seed oils (mg/100 g).

	α-T	α-T3	β-T	γ-T	P-8	γ-T3	δ-T
Linseed	0.8	n.d.	n.d.	39.0	13.5	n.d.	0.5
Apricot (Sweet)	2.8	n.d.	n.d.	67.3	n.d.	n.d.	2.2
Apricot (Bitter)	3.1	n.d.	n.d.	81.0	n.d.	n.d.	2.5
Pear (Ankara)	5.4	n.d.	0.2	55.6	0.6	n.d.	1.3
Fennel (Dulce)	n.d.	1.4	n.d.	0.5	n.d.	18.2	n.d.
Peanut	14.9	n.d.	0.5	16.9	n.d.	0.5	0.7
Apple (Golden)	51.4	n.d.	28.3	6.8	n.d.	n.d.	3.5
Apple (Starking)	54.4	n.d.	30.9	0.5	n.d.	n.d.	1.7
Cotton	36.2	0.3	0.2	48.7	1.1	n.d.	0.3
Quince	49.6	3.2	27.8	8.2	n.d.	n.d.	5.2
Chufa	68.5	n.d.	1.4	0.5	0.3	n.d.	n.d.
Apple (Starking)	60.5	0.3	34.3	n.d.	n.d.	n.d.	n.d.

n.d., not detectable; T, tocopherol; T3, tocotrienol; P-8, Plastochromanol-8.

All the oils investigated exhibited differences in their tocopherol contents. The predominant tocopherol compounds in the different seeds oils were α- and γ-tocopherol. Additionally in some oils lower amounts of α-tocotrienol, β-tocopherol, plastochromanol-8 and δ-tocopherol were found. The main vitamin-E-active compound of fennel seed oil was γ-tocotrienol with 18.2 mg/100 g. Also seed oils of other members of the family *Apiaceae* are characterized by a higher content of tocotrienols, such as dill (*Anethum graveolens)* (10.2 mg/100 g α-tocotrienol) or coriander (*Coriandrum sativum* L.) (23.1 mg/100 g γ-tocotrienol) [33].

The α-tocopherol group comprised of apple (golden) (51.4 mg/100 g), apple (starking) (54.4 mg/100 g, 60.5 mg/100 g), quince (49.6 mg/100 g) and chufa (68.5 mg/100 g) seed oil. Except for chufa seed oil, these oils also contained higher amounts of β-tocopherol with an average amount of about 30 mg/100 g ranging between 27.8 (quince seed oil) and 34.3 mg/100 g (apple seed oil (starking)). γ-tocopherol was predominant in linseed oil (39.0 mg/100 g), apricot seed oil (sweet, 67.3 mg/100 g), apricot seed oil (bitter, 81.1 mg/100 g), pear seed oil (55.6 mg/100 g), and cottonseed oil (48.7 mg/100 g).Among theseed oils in this group only cottonseed oil contained considerably high amount of α-tocopherol (36.2 mg/100 g) while for the other seed oils γ-tocopherol accounted for between 72% and 93% of the total vitamin-E-active compounds. In linseed oil remarkable amounts of plastochromanol-8 were found (13.5 mg/100 g). Peanut oil neither belongs to the α-tocopherol nor to the γ-tocopherol group. The oil showed the lowest total amount of vitamin-E-active compounds of all investigated oils with almost equal amounts of α-tocopherol (14.9 mg/100 g) and γ-tocopherol (16.9 mg/100 g).

## 4. Conclusions

Linseeds, apricot seeds, pear seeds and peanut seeds contained more than 30 g/100 g oil making them interesting as valuable sources for oil production. Due to the lower oil content the other seeds can be interesting for oil processing if the raw material also contains protein with valuable amino acid composition, such as soybeans. The value of the oil depends on the fatty acid composition and the composition of vitamin-E-active compounds. Here the oils can be divided into two groups, one predominant in oleic acid with contents higher than 50 g/100 g (apricot seed oil, peanut oil, chufa seed oil) and one group mainly comprised of linoleic acid (pear seed oil, apple seed oil, cottonseed oil and quince seed oil). Linseed oil is characterized by a high amount of α-linolenic acid and fennel seed oil contained higher amounts of 18:1 fatty acids with petroselinic acid as predominant fatty acid which is usable in technical applications. Especially the fatty acid composition of apricot seed oil with low amount of saturated fatty acids, high amounts of oleic acid and moderate amounts of polyunsaturated fatty acids seems to be an interesting sources as edible oils for human nutrition. The composition of vitamin-E-active compounds of the different oils is characterized by either high amounts of α-tocopherol together with moderate amounts of β-tocopherol or by high amounts of γ-tocopherol. The predominant vitamin-E-amount in fennel seed oil is γ-tocotrienol and peanut oil contains more or less equal amounts of α- and γ-tocopherol. High sources of vitamin-E-active compounds with more than 70 mg/100 g are apricot seed oil, apple seed oil, cottonseed oil and quince seed oil.

In summary it can be concluded that raw materials such as seeds from fruit and vegetable processing are interesting and valuable sources for the production of vegetable oils usable as suppliers of fatty acids and vitamin-E-active compounds in human nutrition or technical applications.
